# Emphysema and Bronchial Dilatation

**Published:** 1881-08-20

**Authors:** Jas. H. Low

**Affiliations:** New York


### EMPHYSEMA AND BRONCHIAL DILATATION.

BY JAS. H. LOW, M. D., OF NEW YORK.

I offer the subjoined article, hoping it may be the means of obtaining from the profession some practical suggestions upon the treatment of a disease which, to me, is so difficult of diagnosis.

I was called, about one month ago, to see Mr. H., who resides on 52d street, in this city, a native of this State, age 41 years. He informed me that he had been sick eleven years, but able, a good deal of the time, to go about the city and attend to his business, which is that of a dairyman; that he has had a number of medical men in this city to attend him, and one in Albany—the latter one pronouncing his left lung destroyed, and all who attended him pronouncing his case consumption, treating him awhile and then discharging the case, telling him to take care of himself and avoid all extremes which is the best course you can pursue. Thus he has passed from one physician to another for the last eleven years, paying out, as he tells me, fifteen hundred dollars doctor's bllls.

This was his verbatim statement to me on my first visit. I told him it was necessary for me to make a thorough examination of his case before I could venture an opinion, to which he readily assented.

*Physical Signs*—There was some dullness of resonance over the upper portion of the left lung on percussion; and on auscultation the crepetant rale, to some extent, with bronchial respiration and bronchophomy; respiration at times cavernous, with respiratory and vocal resonance; great dyspnoea; expecteration of sputa resembling a small raw oyster. Considerable expectoration or vomiting of blood, which I was led to believe, from the quantity and dark color, was from the stomach, and was hæmatemesis and not hæmoptysis, as was supposed by most of his former medical attendants.

The dyspnoea is one of the prominent symptoms in this case, and while the patient is quiet he feels as though he could take any amount of exercise with impunity, but a little exertion on his part develops this symptom, than which there is none other more distressing to him. I am satisfied in making out my diagnosis that my patient has emphysema or dilatation of the pulmonary air cells of the left lung; at least I am satisfied that there exists some bronchial dilatation, and I attribute the dullness on percussion referred to above to condensation of the lung around the expanded part. I thought also that his heart was slightly enlarged, with some valvular change from the dyspnoea and accelerated circulation on the slightest exercise. He complained, also, of considerable uneasiness, amounting to a dull pain in the small of his back, pain increasing considerably when bending over, with numbness in the lower extremities when sitting a short time, and a partial loss of sensibility and a disinclination to move. I thought that he was threatened with (if I may be allowed to use the term) reflex paraplegia, but supposed that the symptoms might arise from relaxation and an enervated condition of his system.

Consumption is not heredetary in his family. He was quite stout previous to his attack. Temperature now about 100, pulse 104. Ænemic in appearance. Weight, 105 pounds; previous to his sickness, 148 pounds: considerable nervous excitability; pain upon pressure in the right hypochonderiac region; heavily coated tongue; but little appetite; great torpor of the liver.

My first object in the treatment of the case was to correct the hepatic derangement. I gave him a pill of blue mass, grs. j; podophyllin, grs. ¼; ext. colocynth comp., grs. j, at bed-time each night, which acted finely. In addition to this, I gave him the ferrated cod liver oil, phos. ferri, grs. ij; cod liver oil, ʒij. three times daily. Subsequently I changed to tinct. ferri. chloridi and cod liver oil. I kept him on this treatment for two weeks; his tongue cleaned off beautifully; appetite much better; sleeps soundly all eight, and says he feels decidedly better. Yet he is harrassed with the dyspnoea. I described his case to Dr. Lockrow, on Madison avenue, who has had considerable experience in throat affections and lung diseases, and he suggested that probably good results might follow from the use of the new remedy, fluid extract of quebracho, and through his kindness I was furnished with a portion of the medicine with instructions how to administer it. It is claimed to be almost a specific in dyspnoea. I commenced using it in small doses—twenty minims twice daily, gradually increasing it two minims daily, until I had reached thirty-two minims, and have fallen back to the first dose, as it seemed to produce some nausea. I am now increasing gradually again daily as at first.

I have been, so far, well pleased with the result, and can safely recommend its use to the profession in the treatment of dyspnoea.

There was about forty-five cases reported at the last meeting of the County Medical Society of New York, a majority reported favorably—all Dr. Lockrow's.

I have now added to mv treatment the following pill: pyrophosphate ferri, grs. j; ext. nux vomica, grs. ¼; sulph. quinia, grs. j, one pill three times daily.

At this writing tongue nearly clean; appetite good; sleep refreshing; pulse, 96 to 100· respiration, 20; dyspnoea disappearing; all the symptoms improving, and the patient says he will get well, certain.

